# Machine learning approach for predicting production delays: a quarry company case study

**DOI:** 10.1186/s40537-022-00644-w

**Published:** 2022-07-16

**Authors:** Rathimala Kannan, Haq’ul Aqif Abdul Halim, Kannan Ramakrishnan, Shahrinaz Ismail, Dedy Rahman Wijaya

**Affiliations:** 1 Department of Information Technology, Faculty of Management, Multimedia University, 63100 Cyberjaya, Selangor Malaysia; 2 Business Development Manager, PETROPRO (Malaysia) Sdn Bhd, 43650 Kuala Lumpur, Malaysia; 3 Faculty of Computing and Informatics, Multimedia University, 63100 Cyberjaya, Selangor Malaysia; 4 School of Computing & Informatics, Albukhary International University, 05200 Alor Setar, Malaysia; 5 School of Applied Science, Telkom University, Bandung, West Java 40257 Indonesia

**Keywords:** Machine Learning, Production delay, Prediction models, Quarry Industry

## Abstract

Predictive maintenance employing machine learning techniques and big data analytics is a benefit to the industrial business in the Industry 4.0 era. Companies, on the other hand, have difficulties as they move from reactive to predictive manufacturing processes. The purpose of this paper is to demonstrate how data analytics and machine learning approaches may be utilized to predict production delays in a quarry firm as a case study. The dataset contains production records for six months, with a total of 20 columns for each production record for two machines. Cross Industry Standard Process for Data Mining approach is followed to build the machine learning models. Five predictive models were created using machine learning algorithms such as Decision Tree, Neural Network, Random Forest, Nave Bayes and Logistic Regression. The results show that Multilayer Perceptron Neural Network and Logistic Regression outperform other techniques and accurately predicts production delays with a F-measure score of 0.973. The quarry company's improved decision-making reducing potential production line delays demonstrates the value of this study.

## Introduction

The mining and quarrying industry is regarded as a potentially substantial contributor to Malaysia’s economy [1]. Perceiving knowledge and learning from data is a major difficulty in industrial organizations, especially those in the quarry and mining industries. Real-time data analytics faces numerous challenges in real-world settings, while a significant amount of legacy, enterprise, and operational data stays untapped [2]. In this research, the case study company has been operating for more than 40 years, providing a firm foundation of quality stones and rocks to all construction works in Malaysia, ranging from road stones, housing constructions, bituminous products, railways, and airport runways. The case company’s bestselling products are high quality aggregate as well as premix products. The success of the company is largely related to their professional expertise and the ability to provide rapid and efficient services to their customers, especially in providing quality granite products at a very competitive price. Consequently, the company is recognised as a progressive and viable business entity, contributing effectively towards the nation's economic development in general and in the states of Negeri Sembilan, Selangor, and the Federal Capital, Kuala Lumpur in particular. In this company, prediction of potential production delays including identification of the causative factors is very important so that it can immediately mitigate and improve company performance.

The goal of this research is to discover potential delays in the quarry company's production so that they can increase operational efficiency by lowering the causal and important elements that affect production time and output. A predictive model was developed to assist and aid the company identify the potential and causation of the delay, therefore offering data-driven decision making in decreasing the prospective delay, based on a research opportunity in the area of machine leaning for prediction of production efficiency. The dataset consists of six (6) months period of production records, which include a total of 20 columns for each production record for two machines, namely Machine 1 (C1008) and Machine 2 (C125). Cross Industry Standard Process for Data Mining (CRISP-DM) approach is followed to build the machine learning models [3]. Five predictive models were built by applying machine learning techniques i.e. Decision Tree [4, 5], Neural Network [6, 7], Random Forest [4, 6], Naïve Bayes and Logistic Regression. The results of the potential production line delay provide insight into operational efficiency.

The rest of this paper is organized as follows. The related works section contains the works of literature related to technological trend for business processes and operations improvement including applications of machine learning techniques for prediction tasks. The research methodology section discusses the CRISP-DM approach to solve this problem. The findings are explained in the Result and discussion section. Finally, we draw the conclusion in the conclusion section.

## Related works

“Data mining and analytics have played an important role in knowledge discovery and decision making in the process industry over the past several decades” [8]. Machine learning serves as a computational engine to data mining and analytics, in which it is used for information extraction, data pattern recognition and predictions. Machine learning techniques have been successfully reported for prediction such as rainfall amount [9], poverty level prediction [10–12], income of campus alumni [13], and COVID-19 related cases [14, 15], etc. Predictive modelling approaches in business process management provide a way to streamline operational business processes [16]. Process mining can discover the process workflows in the company, activity actions, and the mechanism of machines [17], as well as allowing the identification or diagnosis of fact-based problems [18]. Process mining explores the discrepancy between data of events, i.e. observed behavior, and models of processes to detect anomalies, compliance checks, predicts delays, facilitates decision-making, and suggest process redesigns [17]. Nevertheless, machine learning algorithms could be adopted into the process mining techniques, in producing predictive analysis and models.

Industry revolution 4.0 emphasizes the use of technology to improve production operations in the manufacturing industry. This drive attracted many academic researchers and experts to focus on applications of machine learning in production operations for fault diagnosis and machine maintenance [19]. Various types of machine learning techniques have been used in prediction of production delay by the existing literature and this study have used four machine learning techniques; Decision Tree, Neural Network, Random Forest and Naïve Bayes which performed better than other supervised learning algorithms. Decision tree and random forest algorithms are commonly used in fault diagnosis and are considered as classification techniques. While decision tree algorithm builds one optimal decision tree model for predicting the target, random forest algorithm builds a number of decision trees and the final prediction is based on the voting of outcome from each decision tree [20]. Depending on the dataset and variables used, the performance of these two techniques vary where decision tree outperforms random forest and the vice versa [4, 21]. Artificial neural network (ANN) technique, on the other hand, is popularly known for their noise tolerance and is capable of diagnosing a predetermined fault type. ANN has been used for fault detection in die-casting industry [22], to predict faults in a blade pitch system [18] and many more in manufacturing industry. Naïve Bayes algorithm is one of the popular machine learning technique used in predictive models because of its efficiency and it can perform well with a small training dataset [23]. In this paper, these four machine learning techniques have been applied to build predictive models to determine the production delays in the case study company.

## Research methodology

**Fig. 1:**
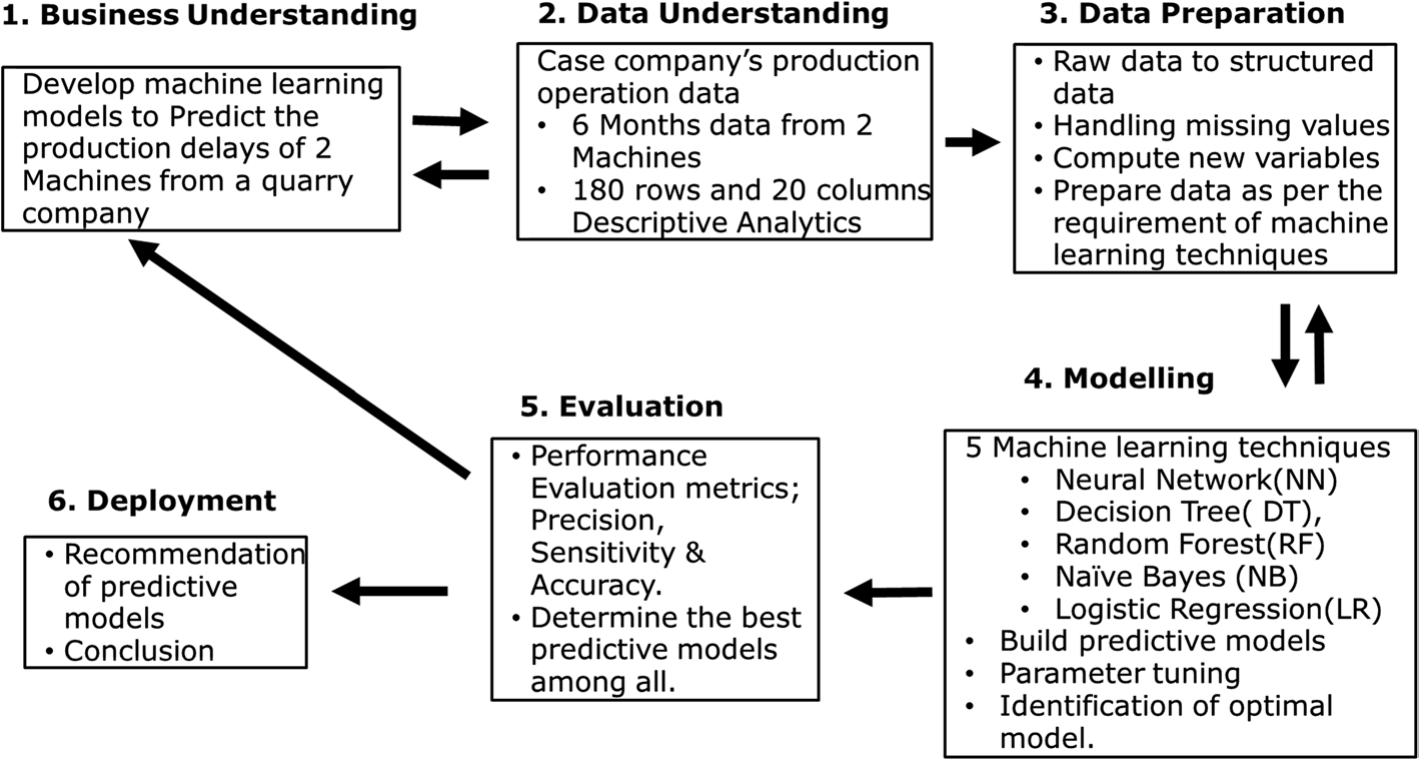
Research methodology based on CRISP-DM

This study adopts CRISP-DM approach to analyze the problem and apply data analytics using machine learning techniques to build predictive model that could be implemented to improve operational efficiency of the production line [24]. Figure 1 shows the phases in CRISP-DM, followed by the details of the research process undertaken in this research.

### Business understanding

The CRISP-DM starts with the Business Understanding phase, which consists of identifying business goal and data mining goal. As mentioned in above, the dataset used for this research came from case study Company that has been operating for more than 40 years. As part of their initiative to improve their overall performance especially in the production and operation sector, the case company is executing a project that requires them to explore on data analytics and machine learning. The source of delay, which can affect the ultimate output and production in days, is a common issue that occurs throughout the manufacturing process. As a result, the business goal is to identify significant delay reasons, which will aid in making manufacturing more efficient and removing the potential and cause of delays.

The purpose of data mining is to use the details of production operations to predict the cause of the delay. To achieve the data mining purpose, four machine learning algorithms are used to develop prediction models: Logistic Regression, Naive Bayes, Decision Tree, Neural Network, and Random Forest. Stratified sampling is utilized in dealing with imbalanced data. To evaluate the performance of the various predictive models, standard metrics such as sensitivity, precision and accuracy are used. KNIME analytics platform, open source data science software was used to carryout data mining process.

### Data understanding

**Fig. 2:**
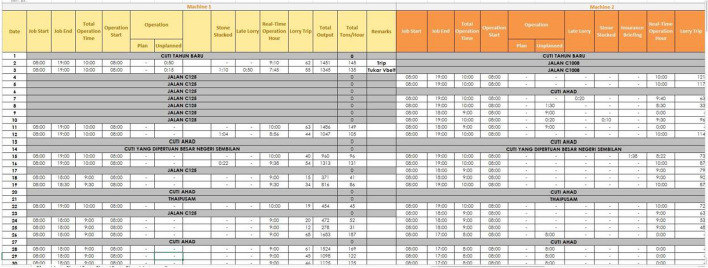
Preview of the production dataset—the raw data

The target dataset was obtained from the Operation Department of the case company containing 180 rows and 24 columns such as Job Start, Job End, Total Operation Time, Operation Start, Maintenance Plan, Maintenance Unplanned, Insurance Briefing, Full Stockpile, Blasting, Pump Cleaning, Out of Stone, Rain, Stone Stocked, Late Lorry, Quarry-Top Full Water, Road Expansion Quarry-Top, Real-Time Operation Hour, Lorry Trip, Total Output and Total Tonnes per Hour. The outcome variable is column “Delay”, which has True / False values indicating whether the production has occurred delay or not in the current production period. Figure 2 below shows the screenshot of the dataset for a few samples before data pre-processing.

**Fig. 3:**
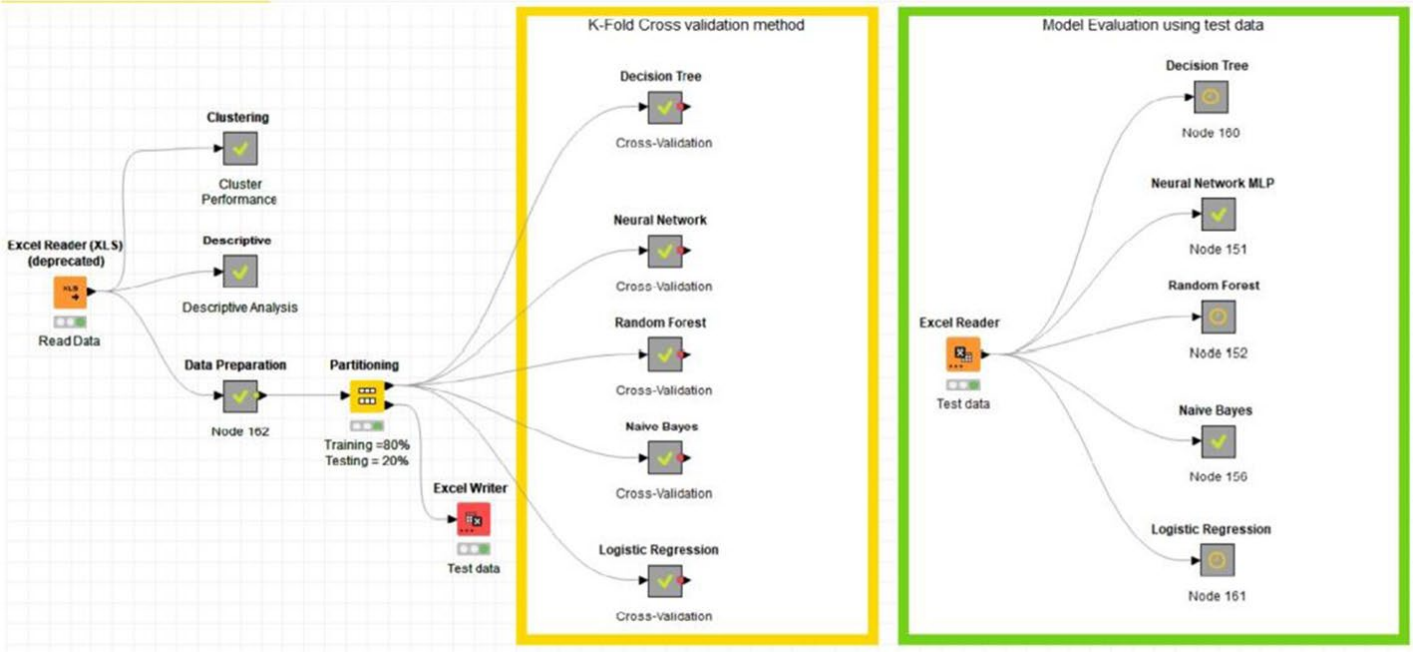
The overall KNIME workflow for the prediction of production delay analysis

### Data preparation

Primarily in Data Preparation phase, this research explored the dataset to see whether the input dataset is standardized and any missing values are observed. In the preliminary process, we observed that the data for each month had different format and was not standardized. Therefore, major preparations were made to standardize all parameters in the data for each month. On the other hand, the dataset has many missing values that were represented by “-” in most delay predictors, in which later was changed to “0” to signify that there is not delay value within the predictor. Besides that, the dataset is segregated into Machine 1 and Machine 2 to different spreadsheets, which later was restructured with added columns labelled “Machine” and “Delay”. Furthermore, the row where the Date falls on a holiday and no production were produced is removed as there is no data input in the dataset. As a result, there are 151 rows and 23 columns of data merged for both machines into one spreadsheet.

Most of the pre-processing work was mainly on formatting the parameters, in which most data input was not in the same category. Prior discussion suggested that the predictor operation time data input was labelled in the unit of hours. However, the input was not standardized as some data was in time format and few in number format. Besides that, the time format is changed to 24 h formatting. The column “operation” was removed as it was observed redundant and overlapping the category of real-time operation. Therefore, it was not used in the prediction model since most of the data under the “operation” column is empty. Apart from that, all the variables were combined into one spreadsheet to ensure that it is readable by the software.

Observing the distribution of number of delays, it is found that 19 occurrences of the production days are delayed due to Maintenance Unplanned and 67 occurrences are delayed caused by Late Lorry. Hence the delay was labeled as two categories where “True” means the production are delayed and “False” means that there is no delay in the production. All the columns were normalized using min–max normalization method as part of the pre-processing to apply neural network technique.

### Modelling

In this step, various machine learning techniques were used to develop predictive models. Logistic Regression, Neural Network, Naive Bayes, Random Forest and Decision Tree are used in predicting the cause of delays in the production days. Naïve Bayes models can produce robust predictions if the predictors have small correlations, even with a simple architecture [8]. Decision Trees are easy to interpret and are capable of giving insights about the important features. Random Forest is an improved version of decision tree, which can produce really good and robust predictions [20]. Artificial Neural Network (ANN) allow complex nonlinear relationships between the target variable and its predictors [18].

Stratified sampling method was used with k-fold cross validation to handle imbalance dataset. Ten numbers of validations are set for training and validation of data [25]. K-fold cross-validation is a resampling method in which the entire data set is partitioned into k sets of almost similar size. The model is trained on the remaining k-1 sets once the first set is chosen as the validation set. After fitting the model to the test data, the test error rate is computed. K-fold cross validation produces a superior model when the data set is small; but, when the data set is huge, it produces no change. This finding is supported by a recent study [25]. Besides that, cross validation helps us to evaluate the quality of the model, facilitating us in selecting the model that will perform the best on unseen data and help avoid overfitting and under fitting of the dataset. Lastly, for the performance evaluation metrics: precision, sensitivity, accuracy and F-measure are calculated to determine which model of the machine learning would give the best results. Figure 3 shows the overall KNIME workflow in predicting the production delay within a manufacturing company. The overall workflow consists of four major parts, which is the descriptive analysis, the unsupervised clustering and developing supervised learning classification models and evaluation.

### Evaluation

In this phase of CRISP-DM, all the machine learning models will be evaluated and compared to select the best model to predict potential delay in the case company production operations. Most commonly used performance evaluation metrics such as accuracy, sensitivity and precision are calculated and compared for all the four machine learning models. Sensitivity and precision are used to make sure the performance of machine learning algorithms, especially to deal with imbalanced data. They can formulate as follows:

1 $$accuracy=\frac{tp+tn}{tp+tn+fp+fn},$$

2 $$\mathrm{sensitivity }=\frac{tp}{tp+fn},$$

3 $$precision=\frac{tp}{tp+fp}$$

where, $\mathrm{tp},\mathrm{tn},\mathrm{fp},\mathrm{fn}$ refer to true positive, true negative, false positive, and false negative, respectively.

F-measure, also known as F1 Score, is a balance of both precision and sensitivity. Hence, this study used F-Score to evaluate the machine learning models.

4 $$F{ - }measure = \frac{{2*{\text{Recall}}*{\text{Precision}}}}{{{\text{Recall}} + {\text{Precision}}}}$$

### Deployment

The best model is recommended for deployment with the insights found from the dataset for data-driven decision making after analyzing the performance of multiple machine learning models using standard metrics; accuracy, sensitivity and precision.

## Results and discussion

### Descriptive analytics

**Fig. 4:**
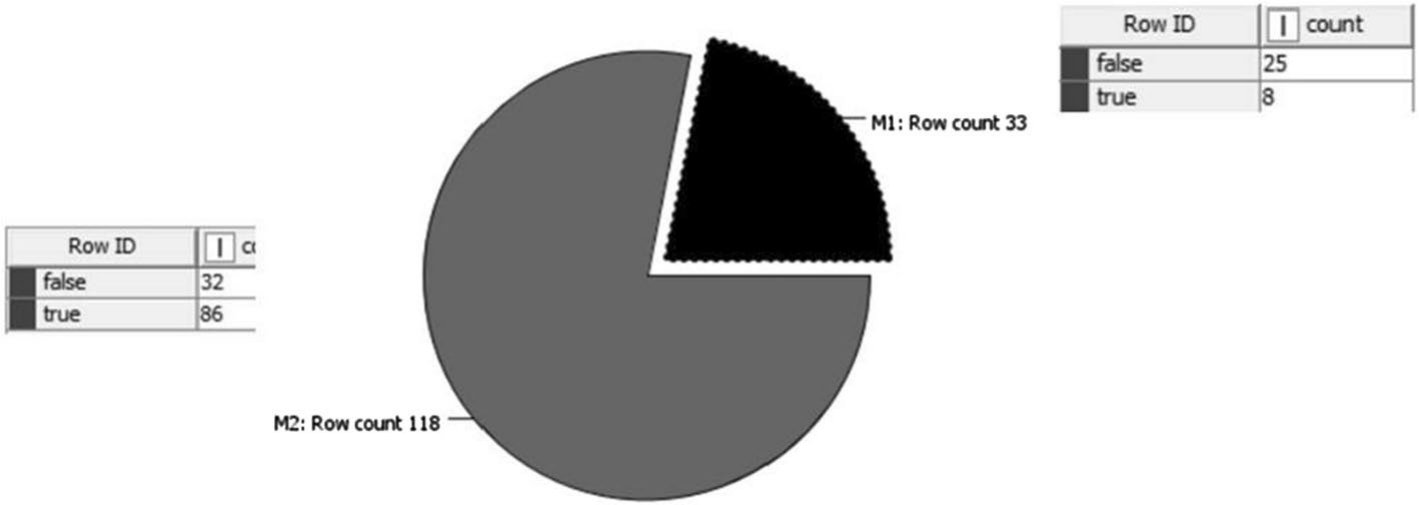
Pie chart on the machine operation occurrences

The dataset consists of 63% of delayed operations and 37% of regular operations. It is observed that Machine 1 was underutilized and Machine 2 was over utilized as shown by Fig. 4. Thus, there are more delay occurred in Machine 2 operation line compared to Machine 1 operation line. In addition to that, the other descriptive analysis conducted is the box plot analysis where the outcome shows the average of total ton per hour is 187.7 whereby the company has to achieve more than the average number as this value is considered as a benchmark value for the company to sustain a good productivity.

**Fig. 5:**
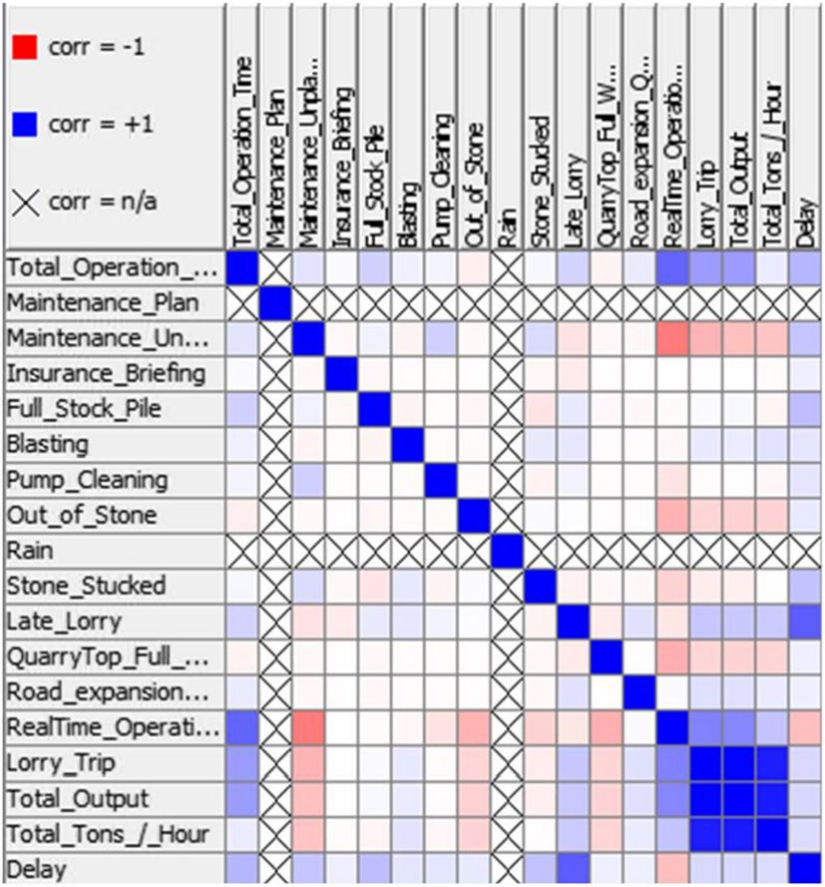
Correlation coefficients of variables

**Fig. 6:**
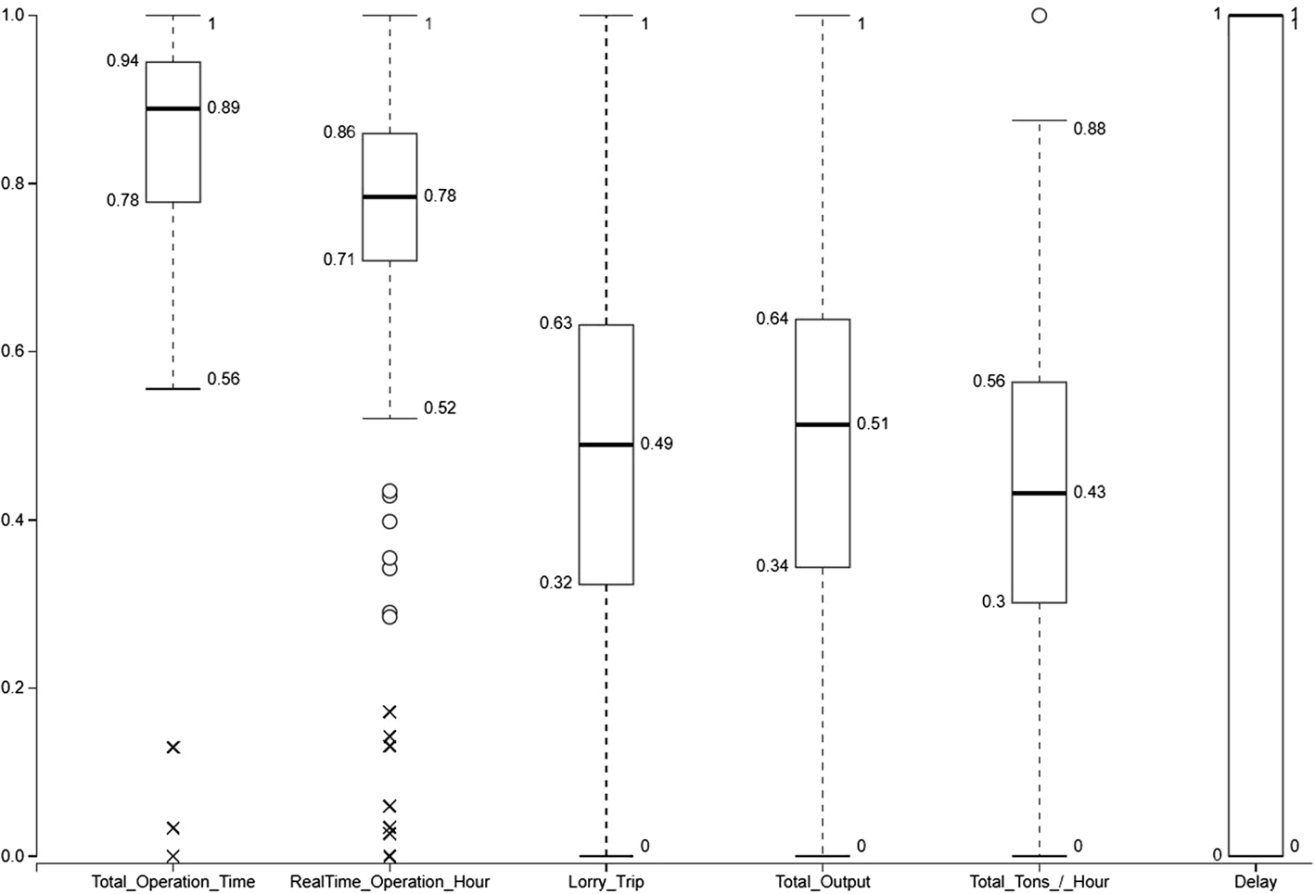
Boxplot illustrating basic statistics and outlier of the production dataset

Besides that, the correlation analysis depicted in Fig. 5 shows that the main factor causing the delay is late lorry, which has a positive correlation to the delay. On the other hand, maintenance unplanned has a significant negative correlation to the real-time operation. These two factors are significant enough for the company to investigate the cause and predict future occurrence as well as prepare a mitigation plan in order to reduce the number of occurrences. Figure 6 boxplot illustrates basic statistics and outlier of the production dataset.

### Segmentation using K-means clustering

It is noticed that the dataset has no obvious segregation of groups, therefore, clustering is required to cluster all data sets in which is deemed fit. For this study, K-mean clustering technique was applied to cluster our dataset [8]. Based on the Silhouette coefficient score, the optimal number for k = 3 was selected. The three clusters are labelled as Low Performance, Medium Performance and High Performance. From the clustering process, the data is segmented into high performance and low performance production which can be viewed through its productivity. Through observation, it is found that the high-performance operation has a higher number of delays compared to the low performance operation, whereby high performance has 67.5% of the occurrence delayed production whereas the low performance only has 50% of the occurrence labelled as delayed production. Therefore, the company would have to prepare themselves should they receive a job that requires a high number of productions.

### Evaluation of machine learning models

The final dataset consists of 151 rows with 18 columns which is further partitioned into 80% training dataset and 20% test dataset. As the data set is small, k-fold cross validation method is used to build the predictive models, in which the number of validations is set to ten (10). K-fold cross validation allows the machine learning process to increase the accuracy of the prediction by learning the concepts from all type of data. In the cross-validation process, the training data is normalized before applying the machine learning algorithm, leaving out the validation dataset. This step ensures that there is no data leakage in the model training. Five supervised machine learning algorithms are selected to predict the production delay which are Decision Tree, Neural Network, Random Forest, Naive Bayes and Logistic Regression. During this phase, the hyperparameters of each algorithm are tuned to identify the optimal models.

The performance evaluation of all machine learning models and the hyper-parameters employed are summarized in Tables 1, 2, and 3. Based on F-measure value, Random Forest exhibited the best performance during the training phase as illustrated in Table 1. However, when all of these models were evaluated with test data, the Neural Network (MLP) and Logistic Regression models emerged as the best prediction models based on F-measure, as shown in Table 2. Both of these models have the same accuracy statistics for predicting the delay: Accuracy = 0.968, Sensitivity = 0.947, Precision = 1 and F-measure = 0.973. Likewise, Decision Tree and Random Forest algorithms produce the second-best models. The Nave Bayes model, on the other hand, is the least accurate.

**Table 1:** Identification of optimal machine learning models from each algorithm

Machine Learning Technique	Accuracy	StdDev	Delay	Sensitivity	Precision	F-measure
Decision Tree (Gain Ratio)	0.963	0.014	False	0.98	0.926	0.952
			True	0.952	0.988	0.957
Decision Tree (Gini Index)	0.956	0.008	False	0.961	0.925	0.942
			True	0.952	0.976	0.964
Neural Network—Multilayer perceptron (Min–Max normalization)	0.904	0.015	False	0.784	0.867	0.86
			True	0.976	0.952	0.927
Neural Network—Multilayer perceptron (Z-score normalization)	0.919	0.015	False	0.941	0.857	0.897
			True	0.905	0.962	0.933
Random Forest (Min–Max normalization, Gini Index)	0.911	0.021	False	0.824	0.933	0.875
			True	0.964	0.9	0.931
Random Forest (Z-score normalization, Gini Index)	0.948	0.013	False	0.941	0.923	0.932
			True	0.952	0.964	0.958
Random Forest (Min–Max normalization, Information Gain Ratio)	0.881	0.008	False	0.745	0.927	0.826
			True	0.964	0.862	0.91
**Random Forest (Z-score normalization, Information Gain Ratio)**	0963	0.008	False	0.961	0.942	0.951
			**True**	0.964	0.976	**0.97**
Naïve Bayes (Z-score normalization)	0.622	0.0	False	0	–	–
			True	1	0.622	0.767
Naïve Bayes (Min–max normalization)	0.378	0.005	False	1	0.378	0.548
			True	0	–	–
Logistic Regression (Min–max normalization)	0.889	0.025	False	0.745	0.95	0.835
			True	0.976	0.863	0.916
Logistic Regression (Z-score normalization)	0.956	0.019	False	0.961	0.925	0.942
			True	0.952	0.976	0.964

Optimal model is denoted with the bold font

**Table 2:** Performance evaluation of optimal Machine Learning models

Machine Learning Technique	Accuracy	Delay status	Sensitivity	precision	F-measure
Decision Tree (Gini Index)	0.935	False	0.917	0.917	0.917
		True	0.947	0.947	0.947
**Neural Network—Multilayer perceptron** **(Z-score normalization)**	0.968	False	1	0.923	0.96
		**True**	0.947	1	**0.973**
Random Forest (Z-score normalization, Information Gain Ratio)	0.935	False	0.917	0.917	0.917
		True	0.947	0.947	0.947
Naïve Bayes (Z-score normalization)	0.613	False	0	–	–
		True	1	0.613	0.76
**Logistic Regression** **(Z-score normalization)**	0.968	False	1	0.923	0.96
		**True**	0.947	1	**0.973**

Optimal model is denoted with the bold font

**Table 3:** Hyper-parameters used in the Machine learning models

Machine learning model	Hyper-parameters
Decision tree	Quality measure/split criterion: gain ratio
	Pruning method: no pruning
Neural network—multilayer perceptron	Number of hidden layers: 1
	Number of hidden neurons per layer: 10
	Maximum number of iterations: 100
Random forest	Quality measure / split criterion: Information Gain Ratio
	Number of models: 100 (static random seed)
Naïve Bayes	Default Probability: 0.0001
	Minimum standard deviation: 0.0001
	Threshold standard deviation: 0.0
Logistic regression	Solver: Stochastic average gradient

In this paper, we compared several algorithms such as Decision Tree, Random Forest, Logistic Regression, Naive Bayes, And Multilayer Perceptron (MLP) As Neural Network algorithm. In this study, Neural Network (MLP) and Logistic Regression surpassed other algorithms with 96.8% classification accuracy. Even though Deep Learning is “state of the art technique” and it has been reported in many case studies [26], in this case study, MLP has shown favorable performance with simpler and faster training processes than the deep learning approach. Hence, we argue that MLP is enough to solve this problem. Moreover, to deal with imbalanced data Stratified sampling method was used with k-fold cross validation. Also, recall (sensitivity) precision, and F-measure were used for evaluation metrics to make sure the performance of machine learning algorithms, especially to deal with imbalanced data. Furthermore, they show satisfactory performance with more than 97% of F-measure values. It means that MLP model has excellent ability to detect each class (delay or no delay) and deal with imbalanced data.

Based on the findings, the following recommendations are proposed to the company: To avoid overusing one machine and causing production delays, the company should use both machines equally and efficiently. The important factors that contribute to low Real Time Operation and Delay include unplanned maintenance and late lorries. This study proposes Neural Network (MLP) and Logistic Regression models to predict the production delay.

## Conclusion

The goal of this research was to use data analytics and machine learning approaches to create prediction models that would help a quarry company's production line run more efficiently. The results were created in the form of descriptive analysis, clustering, and predictive models using five machine learning techniques: Decision Tree, Neural Network, Random Forest, Naive Bayes and Logistic Regression. In a nutshell, this research compares the efficiency of the production line that goes through two main machines, Machine 1 and Machine 2, to identify the potential delay in the production. Considering various factors made available in the dataset, it is found that Neural Network and Logistic Regression, give the best performance of machine learning models in predicting operation delay, with a high score of 0.968 accuracy and F-measure score of 0.973. Thus, Neural Network and Logistic Regression prediction models are recommended for this case study, in which a quarry company could use to provide further decision-making analysis and to strategize an improvement plan to reduce potential delays in production line. The limited dataset, with only 6 months of production data and a small number of attributes, is a limitation of this work. More features, such as the number of workers, downtime, and other relevant data, should be included in future studies to improve the model.

## Data Availability

The data was collected from the case company and is not available to the general public. The authors' data are, however, available upon reasonable request and with the permission of the case study company.

## Abbreviations

CRISP-DM: Cross Industry Standard Process for Data Mining
ANN: Artificial Neural Network
